# RGMQL: scalable and interoperable computing of heterogeneous omics big data and metadata in R/Bioconductor

**DOI:** 10.1186/s12859-022-04648-4

**Published:** 2022-04-07

**Authors:** Simone Pallotta, Silvia Cascianelli, Marco Masseroli

**Affiliations:** Dipartimento di Elettronica, Informazione e Bioingegneria, Via Ponzio 34/5, 20133 Milan, Italy

**Keywords:** Heterogeneous omics big data, Data scalability, Distribution transparency, Tertiary data analysis

## Abstract

**Background:**

Heterogeneous omics data, increasingly collected through high-throughput technologies, can contain hidden answers to very important and still unsolved biomedical questions. Their integration and processing are crucial mostly for tertiary analysis of Next Generation Sequencing data, although suitable big data strategies still address mainly primary and secondary analysis. Hence, there is a pressing need for algorithms specifically designed to explore big omics datasets, capable of ensuring scalability and interoperability, possibly relying on high-performance computing infrastructures.

**Results:**

We propose RGMQL, a R/Bioconductor package conceived to provide a set of specialized functions to extract, combine, process and compare omics datasets and their metadata from different and differently localized sources. RGMQL is built over the GenoMetric Query Language (GMQL) data management and computational engine, and can leverage its open curated repository as well as its cloud-based resources, with the possibility of outsourcing computational tasks to GMQL remote services. Furthermore, it overcomes the limits of the GMQL declarative syntax, by guaranteeing a procedural approach in dealing with omics data within the R/Bioconductor environment. But mostly, it provides full interoperability with other packages of the R/Bioconductor framework and extensibility over the most used genomic data structures and processing functions.

**Conclusions:**

RGMQL is able to combine the query expressiveness and computational efficiency of GMQL with a complete processing flow in the R environment, being a fully integrated extension of the R/Bioconductor framework. Here we provide three fully reproducible example use cases of biological relevance that are particularly explanatory of its flexibility of use and interoperability with other R/Bioconductor packages. They show how RGMQL can easily scale up from local to parallel and cloud computing while it combines and analyzes heterogeneous omics data from local or remote datasets, both public and private, in a completely transparent way to the user.

**Supplementary Information:**

The online version contains supplementary material available at 10.1186/s12859-022-04648-4.

## Background

The rapid progress of Next Generation Sequencing (NGS) technologies and the improvement of data processing pipelines have lead to a dramatic increase in the volume of available omics data with associated high-level features. Both world-wide consortia and private research groups are gathering a huge number of different omics collections [1–7]. A crucial point is to make sense of this amount and variety of omics data, using proper analyses and bioinformatic pipelines to investigate multiple biological and clinical conditions and possibly answer complex issues.

To this aim, suitable big data algorithms as well as integration and processing strategies are fundamental to guarantee scalability and performance, through efficient implementations on high performance computing infrastructures such as clouds, CPU clusters and network infrastructures. Omics datasets are in fact collected within many and heterogeneous data files, structured to trace genomic regions; these files are usually distributed on different repositories and frequently lack of an attribute-based organization or a systematic description of their metadata. Thus, to take advantage of them, available tools for standard knowledge extraction are often inefficient or inappropriate. Even when they have powerful features, rough programmatic interfaces make them not well-suited for biologists and scientists in the biomedical field.

Furthermore, cloud-based approaches and big data algorithms for computational genomics have so far been mainly targeted to speeding up NGS primary and secondary analysis. So, they are focused on read alignment, mapping and feature calling [8–12], while have been rarely directed to tertiary analysis [13–15]. Tertiary investigations aim to extract biological knowledge, like discovering how different genomic regions and their products interact with each other under given clinical conditions. Hence, they require multi-sample seamlessly integrated processing and analysis of region data and metadata from heterogenous omics datasets.

The GenoMetric Query Language (GMQL) [16–18] is a high-level, query language that addresses this kind of tasks performing efficient operations over genomic data and their metadata. Indeed, through parallel computation on cloud-based technologies, it performs implicit iterations over thousands of samples, hosted in its open cluster-based repository. GMQL is thus designed for high scalable performance on large datasets; yet, it supports only batch interactions (via its Web interface or Scala API), requires users to write queries compliant to its syntax, and does not provide direct support for data analysis and visualization.

We developed RGMQL to bridge this gap between the declarative nature of GMQL and the procedural workflows dealing with omics data, commonly carried out in the R/Bioconductor environment. RGMQL is indeed a R/Bioconductor [19, 20] software package able to bring query expressiveness and computational efficiency of GMQL within an interactive data processing flow. It is built over the GMQL data management and computational engine to provide a set of specialized functions that extract, combine, manipulate and compare genomic data and metadata, from both local and remote sources, without requiring any knowledge of GMQL syntax. More important, it is designed to offer complete interoperability and take full advantage of the other packages of data processing, statistical analysis, machine learning and visualization available within the R and Bioconductor frameworks. Notably, it provides extensibility over the most commonly used genomic data structures and processing functions, as to be easily used by researchers used to R programming.

RGMQL can even offer processing outsourcing, i.e., it can assign the analytical computational burden of a processing to a remote GMQL service. Also, it provides data distribution transparency; accordingly, data are always automatically handled and moved based on the actual processing unit (local or remote), without any further user concern. So, RGMQL can easily scale from local to parallel and cloud computing, while processing both local and remote omics datasets in completely transparent way to the user. Notably, data under analysis can be public or private, since when private data are processed remotely they are automatically uploaded in a private area of the GMQL repository, accessible to the proprietary user only.

Hence, RGMQL provides researchers with a valuable ally for omics data tertiary investigation. Being fully integrated within the R/Bioconductor framework, it can straightforwardly cooperate with other packages while it makes really easy to take advantage of GMQL functionalities. Also, it offers GMQL computational facilities and public datasets stored remotely, all ready-to-use for analyses that can involve user proprietary data as well. Furthermore, RGQML is able to guarantee FAIR principles [21] (findability, accessibility, interoperability and reusabilty) not only at the data level, but also at the implementation level. The package can be found in Bioconductor and its code in the associated GitHub project. Genomic data and metadata are easy to find using RGMQL functions to explore the content of the remote repository. Data of interest can be materialized in the local file system and within the R environment to be further processed, ensuring interoperability with proprietary data and reusability. The same interoperability and reusability are provided by the package itself, which can cooperate with other R packages while reuses and extends existing R infrastructure and functions.

### Related works

The analysis of high-throughput heterogeneous genomic data has critically become dependent on robust and efficient bioinformatics approaches. Although many steps ahead have been made in the design of software and pipelines for such data processing, mainly addressing region data usually stored in BED (Browser Extensible Data) format, many software suites are still thought to be used on single experimental files; this is the case of the well-known BEDTools [22] and BEDOPS [23], which are Unix-based command line tools providing manipulation primitives for BED file analysis. Conversely, the GMQL system [16–18] allows implicit and efficient iterations over all the experimental samples of a dataset of interest; in [17, 24] functional and performance comparisons of GMQL with BEDTools and BEDOPS are respectively provided. Furthermore, only the GMQL system has another crucial added value: it is able to handle and perform complex queries and data processing based on both genomic region data and metadata, which are fully organized and supported within the well-defined structure of the Genomic Data Model adopted in the GMQL system.

Despite GMQL original system is certainly the main work related to RGMQL, which inherits all its mentioned strengths, GMQL and RGMQL have highly divergent features. In particular, GMQL only provides batch interactions through its Web application and Scala API, whereas RGMQL works in a continuous R processing flow and ensures full interoperability and analysis of all produced results with the other R/Bioconductor software packages. Although a programmatic interface of GMQL, called PyGMQL [25], has been developed for Python programming language, the possibility of taking advantage of GMQL in a seamless and fully integrated way within the R/Bioconductor environment is of key interest for the bioinformatic community. In fact, Bioconductor [20] is one of the most used open-source software frameworks for open-development and execution of bioinformatic pipelines and omics data analyses [26], which are highly facilitated by the use of R programming language and the availability of many dedicated R/Bioconductor software packages.

Well suited for the analysis and comprehension of high-throughput genomic data [26, 27], Bioconductor strongly encourages extensive reuse of the infrastructure provided by its existing packages, as to enhance interoperability and full compatibility of each library, beyond offering robust high-quality code for data processing and analysis. Its last release (3.14)^1^ includes 2,083 software packages and numerous annotation and experimental data packages from published works. Particularly, the *GenomicRanges* and *GenomicFeatures* [28] packages are the core of the R/Bioconductor infrastructure for omics data handling: they provide scalable data structures for representing annotated ranges (i.e., regions) on the genome and efficient algorithms for overlap computing, coverage calculation, data extraction and other intra- and inter-range operations. Specifically, GenomicRanges was built to include biologically relevant features upon the *IRanges* class, which represents a general vector of ranges. Its *GRanges* class defines a GRanges object that indeed contains an IRanges one, where ranges are enriched with sequence name (e.g., chromosome name), strand information, sequence length and possibly additional region metadata. Furthermore, its *GRangesList* class is designed to build a data structure grouping together GRanges of the same genome and sharing the same region metadata. Conversely, GenomicFeatures offers methods for extracting and manipulating genomic data annotations in GRanges and GRangesList objects. Beyond direct computational facilities, this core infrastructure mostly supports an increasing amount of other Bioconductor packages, including libraries for sequence analysis, differential expression analysis and data visualization. Yet, these kinds of processing require data to be available in main memory: though GRangesList have additional built-in data compression to cope with this issue, the need of RAM memory resident data and results represents undoubtedly a limit for scalability to big data contexts.

In this scenario, we designed RGMQL to be fully integrated in the R/Bioconductor framework and to seamlessly extend its functionalities, with any RGMQL resulting dataset that can be cast to a GRangesList data structure. Not only it ensures interoperabilty with the other R/Bioconductor packages, but also it provides cloud-based computational scalability and efficiency, extending the capabilities of R/Bioconductor that does not directly support scalable genomic data processing on remote clusters. In fact, although Bioconductor and also the general-purpose Comprehensive R Archive Network (CRAN) [19] provide some efficient data manipulation packages used also for genomic data processing, their computational performances are usually enhanced through optimizations, local parallelism or calling lower-level code at run-time. Particularly, the well-known *dplyr* package [29], including many useful data manipulation functions (e.g., filter(), select(), arrange()), provides fast performances thanks to embedded key code pieces written in C++ programming language. Only recently, the *sparklyr* [30] package has been developed to take advantage of the strength of Apache Spark clusters [31], providing compatible back-end for dplyr functions. Nonetheless, to the best of our knowledge RGMQL is the only R/Bioconductor package specifically tailored to comprehensively query heterogeneous genomic data, regardless if locally or remotely located, within a processing context extensible towards parallel computations on cloud-based technologies, even outsourcing the required computing power.

## Implementation

RGMQL is a R/Bioconductor package developed to make available GMQL operators in the R/Bioconductor environment, while ensuring full integration within such a programmatic framework. It is freely available both at https://github.com/DEIB-GECO/RGMQL and at https://www.bioconductor.org/packages/release/bioc/html/RGMQL.html, together with its complete documentation and its vignette, with some task-oriented examples of the package functionalities. Currently, in its first 4 years of life it counts more than 3,800 downloads only from Bioconductor.

Here, after a brief description of the GMQL system and of its data model, RGMQL design is thoroughly discussed, with particular attention to its strengths: the full integration within the R/Bioconductor framework and the distributed processing environments.

### GMQL and its genomic data model

The GenoMetric Query Language [16–18] is a high-level, declarative language developed to efficiently process huge omics datasets and their metadata. Indeed, it expresses operations through compact queries that implicitly imply iterations over all samples. As its name suggests, GMQL is able to process one or multiple datasets based on distal predicates, i.e., conditions related to the genomic distance (in base pairs) between any involved pair of genomic regions; yet, it is also able to support metadata predicates, concerning experimental and clinical properties. Thus, GQML extends conventional operations of relational algebra (e.g., SELECT, PROJECT, UNION, etc.) with further operations specifically designed for genomics, like the domain-specific operations JOIN, COVER, MAP or EXTEND. In Table 1, all GMQL operators are reported and briefly described, together with their corresponding RGMQL functions.

**Table 1:** Genometric RGMQL functions with their extension over already existing R functions and mapping to corresponding GMQL operators

R package of origin	RGMQL function	GMQL operator	Brief description
dplyr	*arrange()*	ORDER	It orders samples sample regions based on metadata region attributes
dplyr	*collect()*	MATERIALIZE	Itsaves persistently the content of any dataset obtained after query completion
dplyr	*filter()*	SELECT	It extracts a subset of samples sample regions using region metadata predicates
dplyr	*group_by()*	GROUP	It groups samples sample regions based on region metadata attributes with the same value
dplyr	*select()*	PROJECT	It selects region metadata attributes to be kept and can update create metadata region attributes
dplyr	*setdiff()*	DIFFERENCE	It discards the regions of the first dataset intersecting regions of the second one
dplyr	*union()*	UNION	It puts together samples of two datasets keeping as region attributes those of the first one
base	*merge()*	JOIN	It returns a dataset by joining the regions of two datasets based on distance region predicates
stats	*aggregate()*	MERGE	It combines all the samples of a dataset into a single sample
–	*cover()*	COVER	It collapses the samples of a dataset into a single sample based on specified rules
–	*execute()*	–	It launches the query execution
–	*extend()*	EXTEND	It generates new metadata attributes for each sample from aggregations applied to region attributes
–	*map()*	MAP	It computes aggregated values from overlapping regions of two datasets

GMQL relies on a formal, unified data description model, called Genomic Data Model (GDM) [24]: it is designed to homogeneously represent semantically heterogeneous omics data and metadata, comprehensively managing the latter ones through a flat attribute-based organization. A GDM dataset is associated with a data schema, where main attributes are fixed (chr, left, right, strand) and represent genomic region coordinates, while all other attributes further characterize each genomic region; conversely, metadata express general properties of each sample and are specified in free attribute-value pair format.

GDM-based datasets are organized, stored and loaded as collections of samples, including both region and metadata files. Specifically, the GMQL project makes available public data of several consortia in an open cluster-based curated repository, including data from The Cancer Genome Atlas (TCGA) [3], the Encyclopedia of DNA Elements (ENCODE) [5] and the 1000 Genomes Project (1KGP) [32]; additionally, any user can have also a personal space to import private datasets, maintaining confidentiality and access control through login sessions.

All datasets can be processed efficiently by GMQL operators through the developed GMQL REST Web Services and Web interface. The underlying architecture ensures scalability and parallelism of processing, and allows using local, remote and distributed File Systems, as well as several deployment strategies on cloud environments or on single Java virtual machines.

The main implementation of the GMQL system, publicly accessible at http://www.gmql.eu/, is installed on a cluster at CINECA, the largest Italian computing centre and one of the most important worldwide. Currently, this GMQL architecture includes an application server and a cluster of machines for execution over the Spark engine and Hadoop Distributed File System. More details about the GMQL system are in [18].

### RGMQL design

RGMQL has been designed to offer both the expressive power of the GMQL query language and straightforward usability by any user with only knowledge of the R syntax. In fact, its functions are developed to directly provide extensibility over core data structures and processing functions commonly used in the R/Bioconductor environment, as well as to be fully interoperable with many other R/Bioconductor packages.

**Fig. 1:**
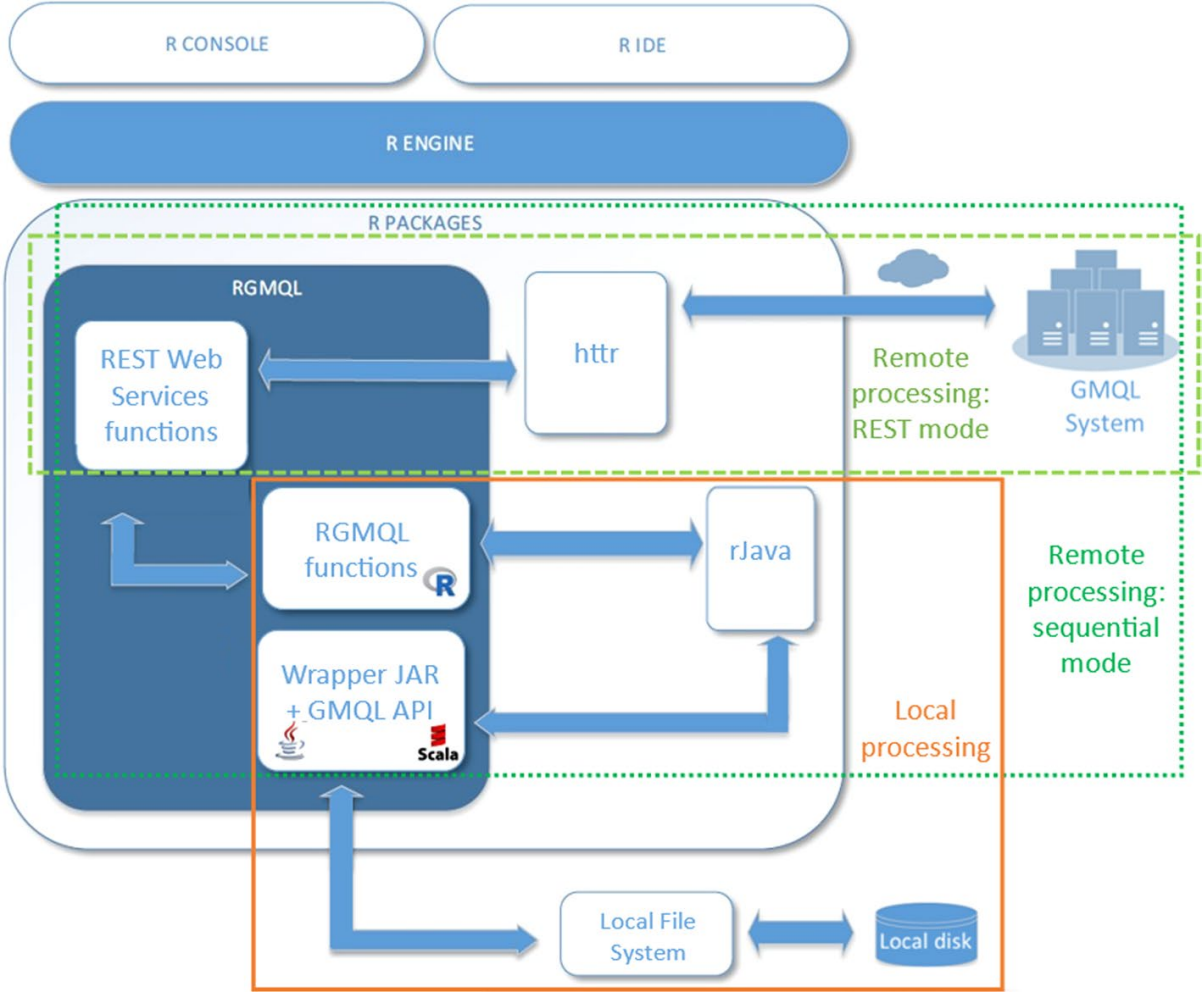
Representation of the RGMQL package within the R/Bioconductor environment. *REST Web services* and *Sequential execution* modules can handle alternative RGMQL processing environments, together with their dependency links to *httr* and *rJava* R packages, respectively

Additionally, it can perform complex and computationally intensive queries (involving region data and metadata of GDM-based datasets) with the same efficiency of the GMQL system. Indeed, it shares the GMQL back-end architecture and cloud-based engine to handle and process in parallel also huge genomic datasets, through remote execution performed at GMQL site. Thanks to the underlying GMQL Apache Spark [33] engine, RGMQL allows scaling up from local to cluster and cloud execution. The back-end interacts with the R front-end through the *Web Services functions* module depicted in Fig. 1, which maps the RGMQL functions to the corresponding GMQL operators (Table 1) implemented in Spark. Furthermore, RGMQL supplies several utility functions (Table 2) needed for seamless integration with the GMQL system, exploration of its curated repository and remote access to the GMQL computational resources.

**Table 2:** Additional RGMQL functions to handle initialization, remote data exploration, processing and result conversions

Function type	RGMQL function	Brief description	Input dataset	Output dataset	Remote processing required
FUNCTIONS TO HANDLE, READ AND ANALYZE LOCAL AND REMOTE DATASETS, PROVINDING ALSO USEFUL CONVERSIONS	delete_dataset()	It deletes a private dataset from remote repository	Remote dataset	–	YES
	download_dataset()	It downloads a private dataset from remote repository to local path	Remote dataset	Local dataset	YES
	download_as_GRangesList()	It downloads a private dataset into R environment as a GRangesList	Remote dataset	GRangesList	YES
	export_gmql()	It creates a GDM-like dataset from a GRangesList	GRangesList	Local dataset	NO
	filter_and_extract()	It filters based on metadata predicates and generates a new GRanges with a chosen list of region attributes. It works if samples have their region coordinates (chr, ranges, strand) in the same order	Local dataset/ GRangesList	GRanges	NO
	import_gmql()	It creates a GRangesList from a GDM-like dataset	Local dataset	GRangesList	NO
	read_gmql()	It reads a GMQLDataset from a dataset (with a valid format) on disk, or from the remoterepository in case of remote processing	Local/Remote dataset	GMQLDataset	YES, if is_local = FALSE
	read_GRangesList()	It reads a GMQLDataset from a GRangeList	GRangesList	GMQLDataset	NO
	sample_metadata()	It retrieves metadata of a specific sample in a dataset	Remote dataset	–	YES
	sample_region()	It retrieves regions data of a specific sample in a dataset	Remote dataset	–	YES
	semijoin()	It supports the filter method defining semijoin conditions on metadata	–	–	NO
	show_datasets_list()	It shows all GMQL datasets in remote repository, both public or privately stored by the user	–	–	YES
	show_all_metadata()	It shows all metadata of a given GMQL dataset either locally or in the remote repository	–	–	NO
	show_samples_list()	It show all samples of a GMQL dataset on the remote repository	–	–	YES
	show_schema()	It shows the region attribute schema of a GMQL dataset on the remote repository	–	–	YES
	take()	It saves as a GRangesList any dataset resulting from local processing. If invoked after collect(), the dataset is materialized also in local File System	GMQLDataset	GRangesList	NO, only for local processing
	upload_dataset()	It uploads a dataset (GDM or not), and a corresponding GMQL dataset is created on the remote repository	Local dataset	Remote dataset	YES
FUNCTIONS TO HANDLE GMQL SERVER AND MONITOR REMOTE JOBS, IF NEEDED	init_gmql()	It initializes and runs GMQL server to execute any processing, and also performs a login to GMQL REST services suite, if needed	–	–	NO
	login_gmql()	Login to GMQL REST services suite as a registered user, specifying username and password, or as guest	–	–	YES
	logout_gmql()	Logout from GMQL REST services suite	–	–	YES
	register_gmql()	Register to GMQL REST services suite	–	–	YES
	remote_processing()	It allows to enable or disable remote processing	–	–	YES
	show_jobs_log()	It shows the log of a specific job	–	–	YES
	trace_job()	It traces a specific job	–	–	YES
	show_job_list()	It shows all jobs (run, succeded or failed) invoked by the user on the remote GMQL server	–	–	YES
	show_queries_list()	It shows all the GMQL queries saved by the user on the remote repository	–	–	YES
	stop_gmql()	It stops the GMQL server processing	–	–	NO
	stop_job()	It stops a specific job	–	–	YES
FUNCTIONS USING QUERIES IN GMQL SYNTAX	compile_query()	It compiles a GMQL query inserted as a text string	–	–	YES
	compile_query_fromfile()	It compiles a GMQL query taken from a file	–	–	YES
	run_query()	It runs a GMQL query inserted as a text string	–	–	YES
	run_query_fromfile()	It runs a GMQL query taken from a file	–	–	YES
	save_query()	It saves into the remote repository a GMQL query, taken from a file	–	–	YES
	save_query_fromfile()	It saves into the remote repository a GMQL query, inserted as a text string	–	–	YES

For each function, we report if it requires remote resources and processing, as well as the formats of its input and output data

Typically, R/Bioconductor packages are developed to handle data of limited size through the use of a single machine; they usually read datasets in RAM memory all at once, and every R object must reside entirely in memory to be processed. This often prevents the analysis of big data, besides requiring long time to process large sets of data. Conversely, RGMQL overcomes such limitations, guaranteeing the same scalability of GMQL and allowing to work with very large datasets, usually not available in local but cloud repositories. Such big data cannot be handled with standard R data structures allocated in the main memory. Accordingly, in RGMQL we have introduced the *GMQLDataset* class, an abstract R structure that represents each GMQL dataset without containing any information about samples (regions or metadata). A R *GMQLDataset* object is indeed only a reference to the desired resulting GMQL dataset, which keeps trace (through a directed acyclic graph) of all the operators to be sequentially applied on the involved input dataset(s) to obtain it. Processing and real availability of the results are actually deferred until the materialize command is called by invoking an execution function. This lazy execution resembles the lazy loading of the R code objects adopted for packages use. It closes the gap between the interactive computations within R/Bioconductor and the typical GMQL batch execution. In fact, it is needed to perform remote execution at the GMQL site, but it is preserved also for local processing since it allows to eventually optimize the processing.

Hence, RGMQL provides a great flexibility of usage in the R/Bioconductor environment, allowing to use indistinctly remote query outsourcing or local processing on the user machine, even working seamlessly on local or remote data. Users are provided with an easy-to-use and interactive framework leveraging both R/Bioconductor and GMQL. Any processing is part of a continuous workflow although RGMQL does not generate any actual result before an execution function is called at runtime, both in local or remote processing. Deferring the computational effort until a result is actually needed (in the local file system or within the R/Bioconductor environment) for further analyses allows to overcome, but synchronize with, the batch-oriented style of the underlying GMQL system.

### RGMQL integration capabilities: interoperability and extensibility

One of the main strengths of RGMQL is to enable a procedural way of working with genomic datasets. Users can in fact perform multiple operations, interleaving RGMQL manipulations with processing and visualizations involving many other R/Bioconductor packages; the only requirement is including each piece of RGMQL code in an initialization-execution block, so that all intermediate and final results of interest are correctly materialized in the main or mass memory and available for further analyses.

**Fig. 2:**
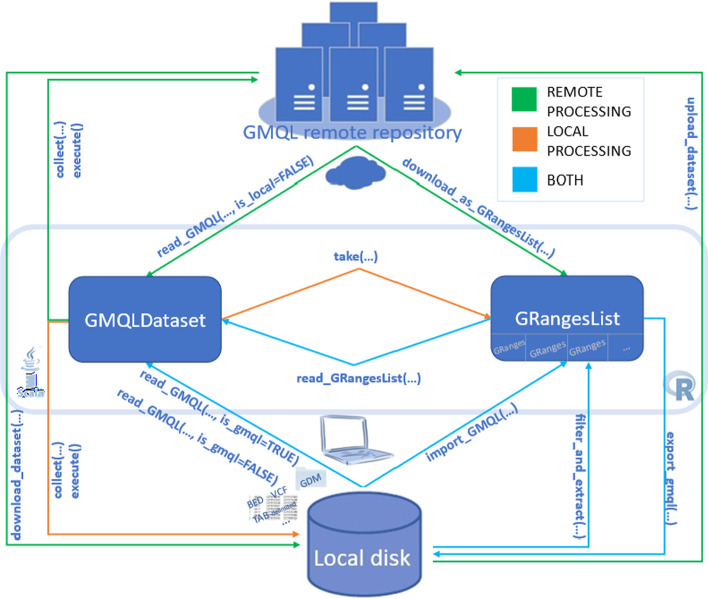
Representation of RGMQL functions for data import/export both locally and remotely. A GMQLDataset is created by the *read_GMQL()* function from a local dataset (in GDM or different tab-delimited format), or from a remote dataset (specifying is_local = FALSE). Any processing is applied on the involved GMQLDataset objects, and the computation and materialization of any result (remotely or locally) is deferred until the *collect()* and *execute()* functions are called. A GMQLDataset can be created also by the *read_GRangesList()* function from a GRangesList. Similarly, a GRangesList can be obtained from a remote dataset through the *download_as_GRangesList()* function, from a local dataset through the *import_GMQL()* function and, in local processing only, directly from a GMQLDataset through the *take()* function

Within such an integrative analytical processing, RGMQL requires full interoperability with the other R/Bioconductor packages. This concerns primarily the dependencies on external packages to convert or bridge idiomatic R constructs and operations to GMQL native syntax. In addition, it greatly regards the needed mapping of GMQL datasets into existing R data structures, suitably and widely-used by other packages in the R/Bioconductor environment. Accordingly, any abstract *GMQLDataset* or any GMQL dataset already saved in mass memory, can be loaded in main memory into a *GRangesList* object, one of the most commonly used R data structure. This makes it accessible in a widely supported format for further processing. Therefore RGMQL is designed to provide not only the efficient data structures, but also several import/export functions that allow data manipulation in *GRangesList* format, regardless the engine chosen to execute the RGMQL processing. An explanatory representation of these functions is reported in Fig. 2, specifying when to apply each of them based on the data source location.

Also, to guarantee full integration and compatibility with the most commonly used R/Bioconductor packages, RGMQL functions were named to override well-known R/Bioconductor processing functions, as to extend their functionalities on *GMQLDataset* instances. In doing this, RGMQL functions move away from the original names of the corresponding GMQL operators, but ease their comprehensibility and application for any scientist used to R/Bioconductor. Particularly, besides a couple of basic functions coming from the *base* and *stats* R packages, RGMQL extends other functions with a well-known meaning that are implemented in the widely-used *dplyr* [29] R package for data manipulation; Table 1 shows the RGMQL functions together with the R package generally defining their functionalities and the corresponding GMQL operators.

Therefore, RGMQL not only ensures interoperability with other packages, but also full integration within the R/Bioconductor environment: indeed, it provides extensibility over well-known functions and genomic data structures commonly used within such a framework.

### RGMQL distributed processing environments: flexibility and scalability

RGMQL can consume computational power directly from the local CPUs/system, operating in the R/Bioconductor local processing environment, or use remote processing resources offered by the GMQL ecosystem. The former option takes advantage of the GMQL possibility to be deployed on a single Java virtual machine, while the others allocate resources and run (respectively only or partially) on a cluster, which is transparently provided by the remote GMQL server. Although local processing is adequate only for limited data size and not suitable for big data processing, it is a relevant added value to be able to work locally. For example, there are sensitive data that users cannot even export in their own private space on the GMQL remote repository, for restriction constraints.

For remote processing, indeed RGMQL lets users log (with private credentials or simply as a temporary guest) into the remote GMQL infrastructure and also manage large public genomic datasets, there hosted in the cluster-based curated repository. Then, it enables users to consume remote GMQL services, providing two ways of accessing a GMQL system instance: directly through the GMQL REST Web services or indirectly, passing through the GMQL Scala API contained into a compressed .jar file enclosed in the RGMQL package.

Hence, RGMQL implements two different sets of functions and processing modes, henceforth referred as: 1) the *REST Web services* mode, which generates HTTP requests, through the *httr* R package, designed to map closely to the underlying HTTP protocol and providing all standard HTTP functions; and 2) the *Sequential execution* mode, which uses the *rJava* R package to interface with the GMQL Scala API (Fig. 1).

The *REST Web services* mode does not require any bridging, since it is executed via Web service functions only. By invoking REST services users can upload local datasets into a private area of the remote repository, compile and run remotely a textual query (written in GMQL syntax and passed as argument of the R code) over remote private and/or public datasets, and download datasets resulting from remote processing into a local folder. All operations can be applied both on originally remote datasets or on local ones previously uploaded; this is directly inherited from the GMQL system approach, which allows the addressed repository to be deployed on a local or a distributed Hadoop File System (HDFS) [34].

The *Sequential execution* mode, instead, lets users work in local or remote processing with both local and remote datasets, as clearly appears in Fig. 1. In local processing, the *Sequential execution* module is only interfaced with the Local File System. Conversely, when users choose remote processing and read any data, the system automatically uploads it on the remote system if it is not already there. Once loaded, RGMQL functions are called to process data remotely, hiding the batch-like interactions that are issued sequentially at execution time. In this case, processing instructions are written as RGMQL functions, which need a direct bridging with the corresponding GMQL Scala API. The bridge is built by implementing the whole logic into a “wrapper” class written in Scala, a dialect of the Java programming language, and using the *rJava* R package to implement conversions on both sides, as reported in Fig. 1. This wrapper class is instantiated in R/Bioconductor and its public methods are called at execution time to run the GMQL operators underlying every RGMQL function. Each method of this wrapper class can indeed directly call the corresponding native GMQL API. However, only primitive data types (integer, numeric, logical and character) are passed with no conversion from R to Scala; all multi-dimensional data types must be converted in Java objects before passing them as arguments to the wrapper class methods. The same applies when implementing the dual logic, passing data back: the conversion is always performed on the R side through *rJava* functions. The RGMQL *execute()* function returns a directed acyclic graph (DAG) including all the GMQL operators to be sequentially applied on the involved dataset(s). A BASE64 serialized version of the DAG is then sent automatically through the direct ’REST Web Services’ path, as to launch the query remotely on the GMQL system.

Notably, in mixed processing the *Sequential execution* module can alternatively process locally both local and remote data (automatically downloaded locally), or ask for outsourced processing of remote or local data (automatically uploaded remotely). Thus, RGMQL offers also data distribution transparency, since data are always automatically handled and moved according to the actual processing unit in use. Regardless of the computing unit, any resulting dataset can be saved (after local processing) or downloaded (after remote processing) in the local File System as a GDM-based dataset. Conversely, when a resulting dataset is still needed in the R/Bioconductor environment, it can be loaded (in local processing) or downloaded (after remote processing) in main memory as a *GRangesList* object, made of a *GRanges* object for each sample and including all its region and metadata attributes. Such kind of loading in *GRangesList* is possible also starting from a GDM-based dataset already in mass memory, as long as the size of the obtained *GRangesList* (or compressed *GRangesList*) object does not exceed the maximum allowed space in RAM.

Overall, the alternative local and remote processing environments offered by RGMQL are easily interchangeable within a single analysis flow. This guarantees high performance scalability, necessary to work on big genomic data, and extreme flexibility of use, as required by the procedural approach of the bioinformatics research, which typically combines different analyses and data sources.

## Results

In the following subsections we illustrate clear and fully reproducible example use cases of usefulness, expressive power and flexibility of the RGMQL package in biologically relevant applications. Their complete workflows are available as R Notebooks in the RGMQL GitHub repository (https://github.com/DEIB-GECO/RGMQL), together with the vignette and reference manual of the RGMQL package. These examples show the processing capabilities of RGMQL in terms of suitability and scalability offered to perform data-intensive computations on large datasets. Also, they demonstrate RGMQL easiness of use within complete workflows: this is enabled by the data distribution transparency, the flexibility in mixing local and remote data and processing modes, and the full interoperability with other R packages when results are imported as *GenomicRanges* objects.

### Use case 1: Mutational analysis of kidney cancer patients

This example use case shows how RGMQL, together with the remote engine and curated genomic data repository provided by GMQL, permits to easily perform genomic analyses on big datasets; particularly, here we take advantage of RGMQL remote processing to analyze somatic mutational events in specific patients affected by Kidney Renal Clear Cell Carcinoma (KIRC), extracted from a large dataset provided by The Cancer Genome Atlas.

First, the datasets in the remote GMQL curated repository are explored using the *show_datasets_list()* function, and available mutation datasets are identified. The *show_all_metadata()* function is then used to find metadata (e.g., age and type of patients) of mutation datasets of interest. In this example we choose the *GRCh38_TCGA_somatic_mutation_masked_2019_10* dataset, containing somatic mutation data regarding 10,187 samples of 33 TCGA cancer types:

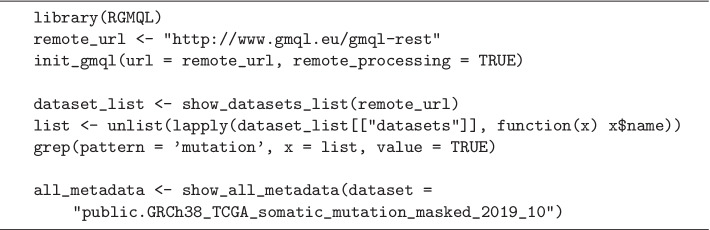



Specific data of interest are then analyzed remotely using the GMQL engine. First the *filter()* function is used to select the KIRC patients of interest, e.g., those younger than 65 years at initial KIRC diagnosis:

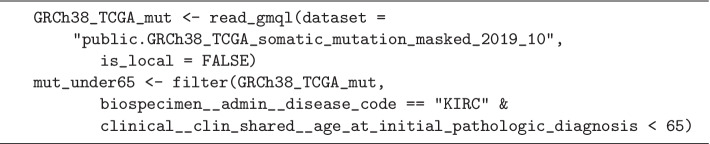



The *map()* function is used to find and count, for each mutation sample and each gene, all somatic mutations occurring in gene regions. Such regions are identified based on the GRCh38 reference genome annotations from the NCBI Reference Sequence database (RefSeq) [35], available in the remote GMQL curated repository:

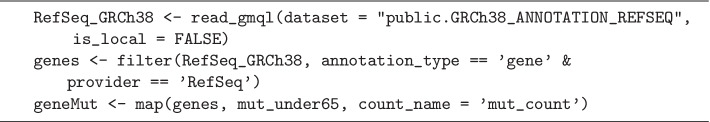



Following, only genes with at least one mutation are preserved through the *filter()* function. Additionally, for each sample the mutated genes are counted, storing such counts in a new metadata attribute called ’geneMut_count’, by means of the *extend()* function:

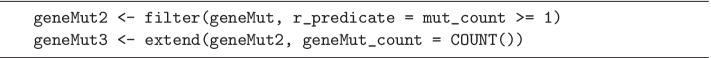



Eventually, found results can be saved in the local file system as a GDM dataset, and also downloaded in memory as GRangesList. The latter ones can be further straightforwardly analyzed and visualized in the R environment with other useful R functions. Particularly, ’geneMut_count’ values can be used to prioritize the samples (one for each patient) from the most to the least mutated one (Additional file 1). From this simple evaluation we can notice that somatic mutated gene counts are quite homogeneously distributed across samples, with a lower quartile of 26 and an upper quartile of 45 mutated genes. Only a sample/patient (sample id: S_00140) clearly appears as an outlier, with its 466 mutated genes. Since genes appear mostly characterized by a single somatic mutation (2 is the 99th percentile), a similar behaviour is obtained also when plotting the total number of mutational events occurring in each patient.

**Fig. 3:**
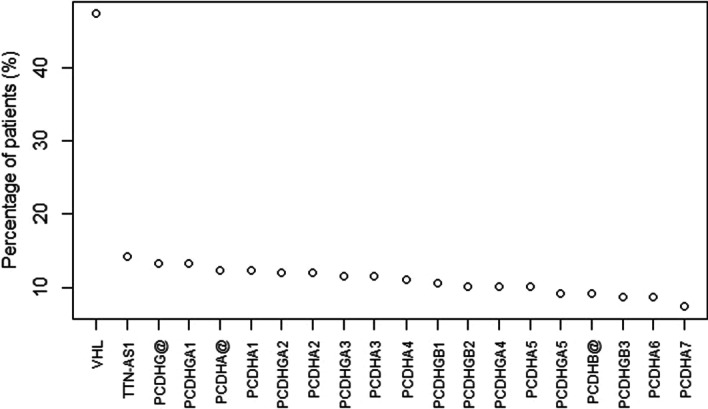
Top 20 genes by percentage of the 217 patients under analysis with the gene mutated

More relevantly, obtained results can be thoroughly analyzed by studying and plotting somatic mutation distributions across samples, as to highlight the most mutated genes in the KIRC population of interest. The mutation count of each gene region is evaluated considering the percentage of involved patients (Fig. 3) or the number of actual mutational events compared to the gene length (Additional file 2). Although in some samples multiple events exist in the same gene region, the top 20 mutated genes are exactly coincident in both cases and include the VHL and TTN-AS1 genes and the PCDH gene family.

The association between the VHL gene and kidney cancer is widely recognized [36–38]: it reaches a DisGeneNet association score of 0.9 [39] and more than 90% of the renal clear cell carcinoma are known to be characterized by somatic mutations in the VHL gene [40, 41]. Conversely, at present the TTN-AS1 gene, encoding a lncRNA transcribed from the opposite strand of the TTN gene, is known to be primarily associated with myopathy and other cardiac and muscular diseases [42].; similarly the protocadherins (Pcdhs), which are predominantly expressed in the nervous system and constitute the largest subfamily of the cadherin superfamily of cell-adhesion molecules, are until now known to be mostly associated with epilepsy and central nervous system neoplasms and disorders [43, 44], but also with several other cancers [41, 44].

**Fig. 4:**
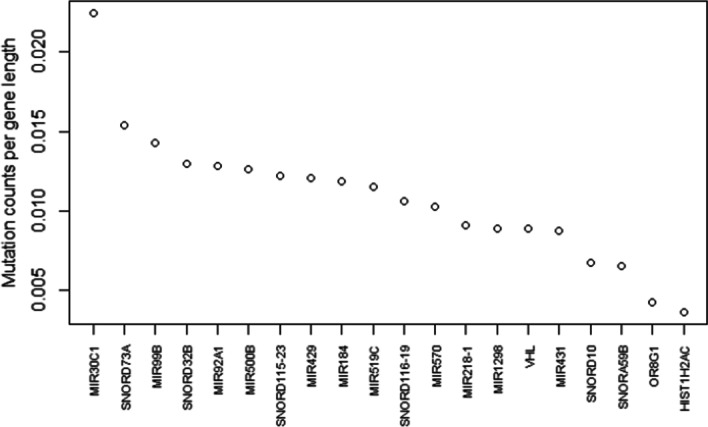
Top 20 genes by number of mutations per gene length across the 217 patients considered

The involvement of VHL gene alterations in kidney cancer is confirmed also when inspecting gene mutation counts normalized by gene length. This normalization determines a top 20 gene list (Fig. 4) including mainly microRNA and small nucleolar RNA genes, favored by their short length (at most about a hundred bases); yet, despite its 12,036 bp length, the VHL gene is still included in this list, together with HIST1H2AC, a gene encoding for a nuclear protein responsible for the nucleosome structure [40], and OR8G1, a gene encoding for an olfactory receptor protein, known to be associated with breast, ovarian and pancreatic cancer [41]. While the evidences on VHL clearly confirm its involvement in kidney cancer, the other found data-driven associations can be worthy of further investigations.

### Use case 2: Patient-wise hierarchical clustering based on combined omics data

In this use case, whose workflow is schematically illustrated in Additional file 3, we show how to combine remote public and local omics data in remote processing, before performing further local analyses on the results. Particularly, we investigate the expression data of both messenger RNA and microRNA genes aligned to the GRCh38 reference genome, focusing on TCGA patients affected by Adrenocortical carcinoma (ACC). The miRNA expression data of such patients are supposed available in the working directory of the local file system in GDM format, within the *GRCh38_miRNA_ACC* dataset (available for reproducibility purpose at https://github.com/DEIB-GECO/RGMQL). Corresponding TCGA ACC expression values from RNA-sequencing experiments are instead extracted from the large *GRCh38_TCGA_gene_expression_2019_10* dataset, available on the remote GMQL curated repository (including 11,092 TCGA gene expression profile samples of 33 cancer types):

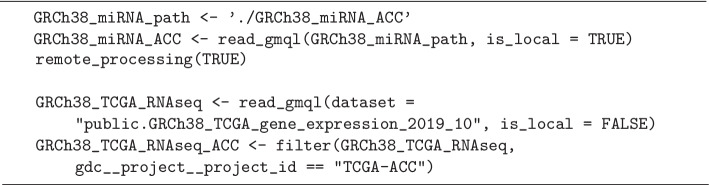



The two resulting datasets are then processed together remotely, after the local dataset automatic uploading, transparent to the user, in a temporary reserved area of the remote GMQL repository. Specifically, the datasets are joined through the *merge()* function based on their *biospecimen__bio__bcr_analyte_barcode* metadata attribute, keeping for each miRNA region also the mRNA gene region at the minimum distance. This is possible thanks to setting to *MD(1)* the *genometric_predicate* and to *BOTH* the *region_output* parameters of the function:

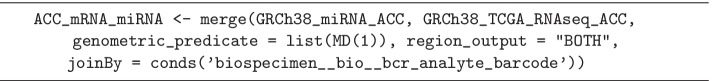



Once the joined dataset is computed and downloaded in the local file system, the remote processing mode can be turned off to proceed with further local analyses. The *filter_and_extract()* function can be used to import only raw count values and gene annotations from the result dataset in the local R environment, within a GRanges object:

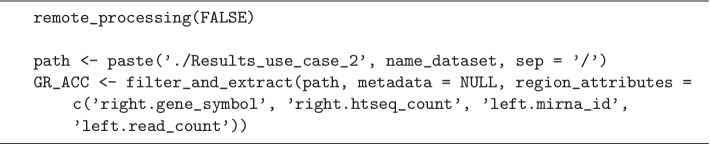



From it, we derive a samples-per-features dataset, where each column hosts values of a different miRNA or mRNA gene and each row is named according to the TCGA ID of the corresponding sample. Differently from the previous use case, here we use the official TCGA patient ID to identify each patient profile, as to ease comparison with preexisting analyses of TCGA data. Such identifiers are collected from the *biospecimen__shared__bcr_patient_barcode* key-value pairs in the metadata table. This table is obtained from the downloaded GDM dataset through the *show_all_metadata()* function, simply as follows:

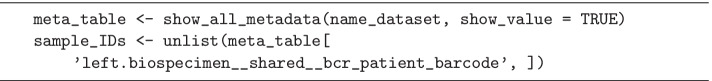



Analogously, we can import in the R environment some clinical annotations of each sample (e.g., tumor outcome status, stage and grade), which may characterize the gene-driven subgroups of patients that emerge from a patient clustering analysis. For this evaluation, we start from a matrix of raw expression values extracted from the previously obtained *GR_ACC* GRanges object. First, we remove miRNA and mRNA genes with null raw values in 25% of samples or more, so as to discard too lowly-expressed genes that are more easily affected by noise and thus less reliable:

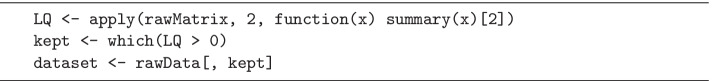



Then, after data normalization performed through the *normalize()* function of the *BBmisc* package [45], we assess and visualize the optimal number of clusters based on the average silhouette width, computed using the *fviz_nbclust()* function of the *factoextra* R package [46] (Additional file 4).

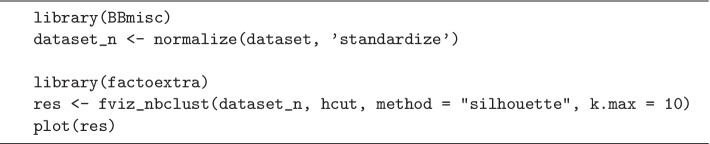



**Fig. 5:**
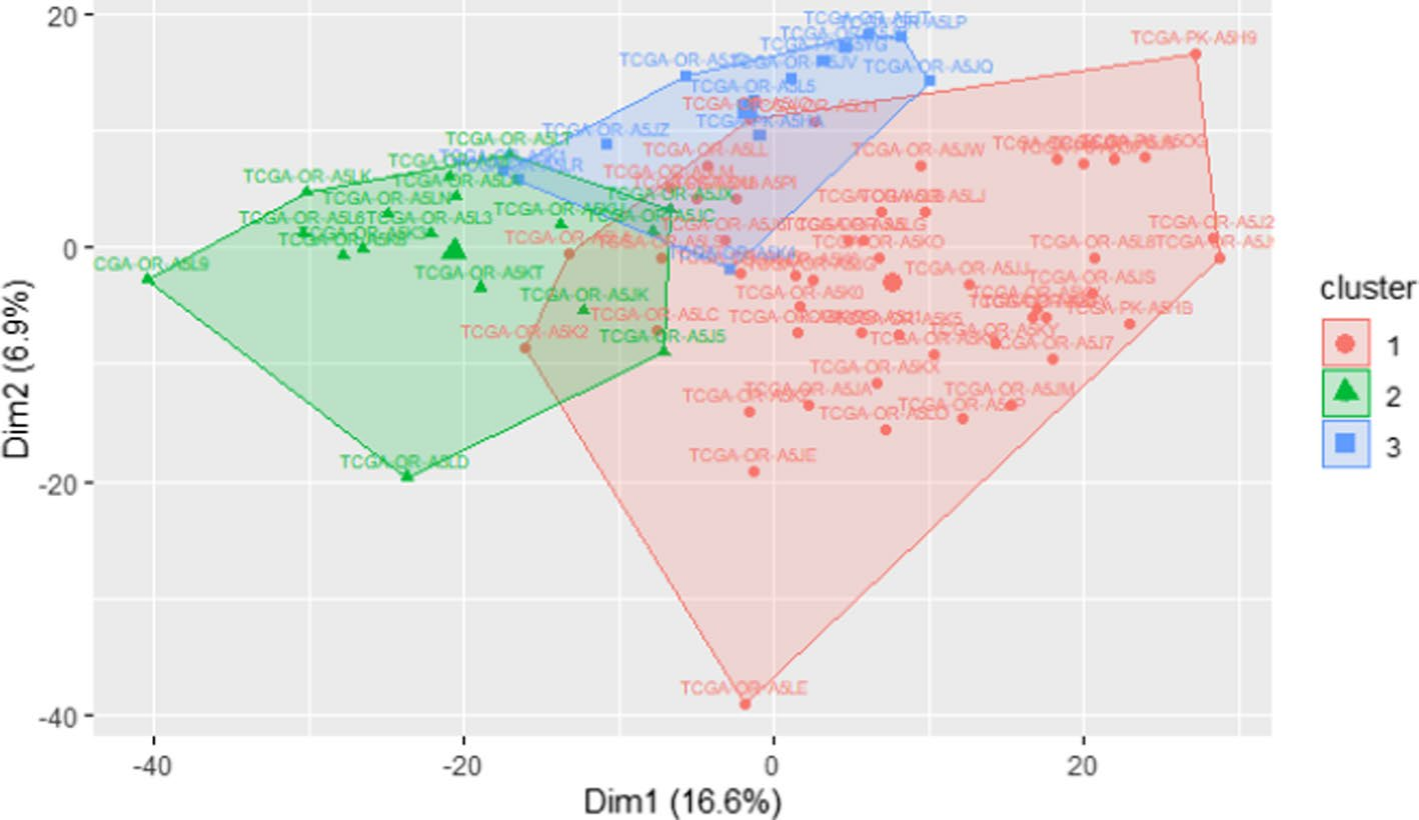
Clusters from patient-wise hierarchical clustering on the first two dimensions of the data principal component analysis. The fraction of variance explained by each dimension is reported as percentage in the corresponding axis label

Considering the found optimal number of three clusters, we apply the Ward Hierarchical Clustering by means of the *hclust()* R function, using the Spearman correlation-based distance as dissimilarity measure among the expression profiles, computed through the *get_dist()* function of the *factoextra* R package; the obtained dendrogram is plotted and the three clusters are highlighted on it with coloured rectangles by means of the *rect.hclust()* R function. Furthermore, the *fviz_cluster()* function of the same *factoextra* R package can be used to depict the three clusters, selected by the *cutree()* R function, in a ggplot2-based visualization [47] (Fig. 5), using as space dimensions the two principal components of the data:

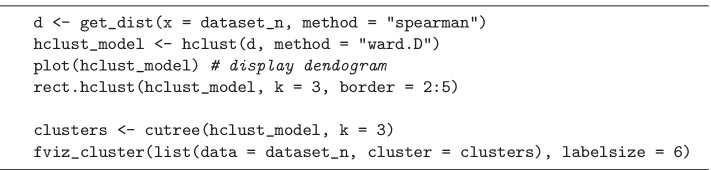



**Fig. 6:**
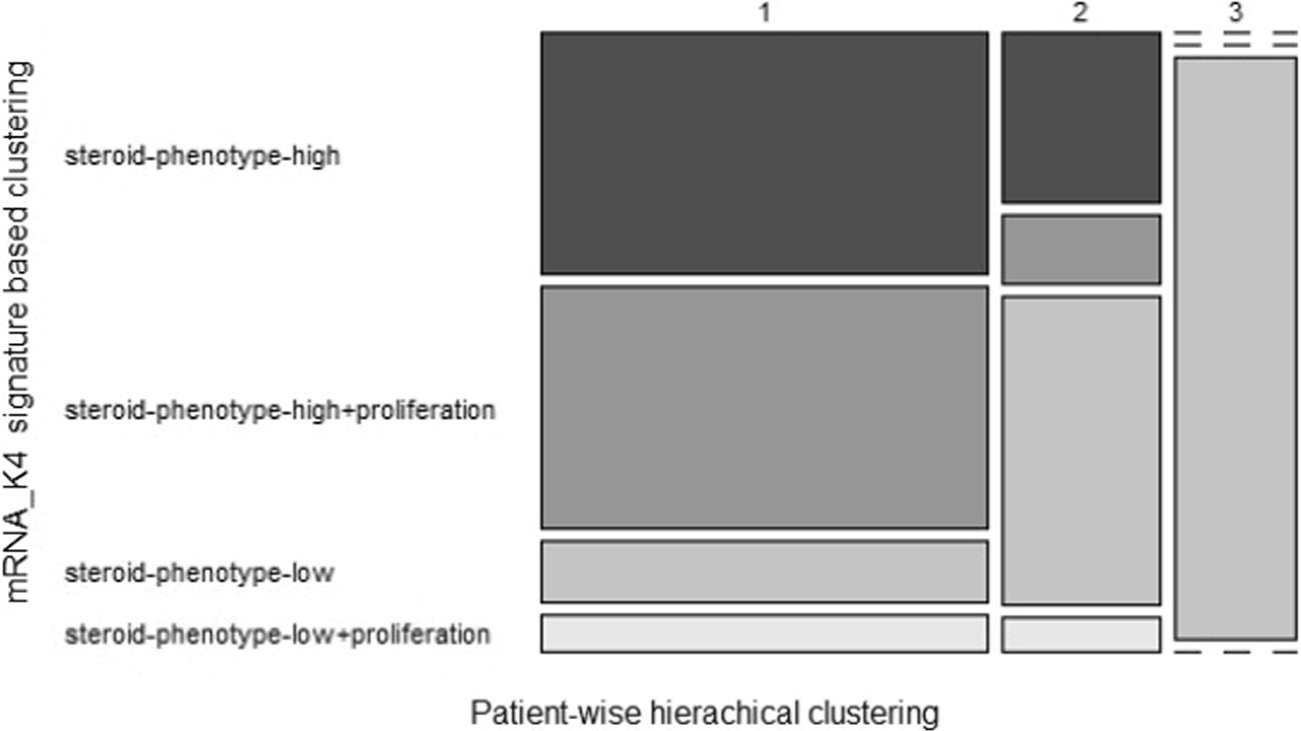
Mosaic plot of the three clusters emerged from patient-wise hierarchical clustering compared with the published clustering results obtained in [48] using the K4 gene signature

The three clusters obtained include 49, 17 and 13 patients, respectively. Each of these clusters can be compared with the previously extracted clinical metadata, as well as with clustering results and survival annotations emerged from the ’Comprehensive Pan-Genomic Characterization of Adrenocortical Carcinoma’ [48] and other pan-cancer studies [49] performed by the TCGA consortium. Accordingly, mosaic plots are useful for showing how different stratification results overlap with each other; particularly, it is interesting to compare our clustering results with those obtained using the K4 gene signature [48], which is indicative of steroid phenotype low and high, with or without proliferation (Fig. 6).

**Fig. 7:**
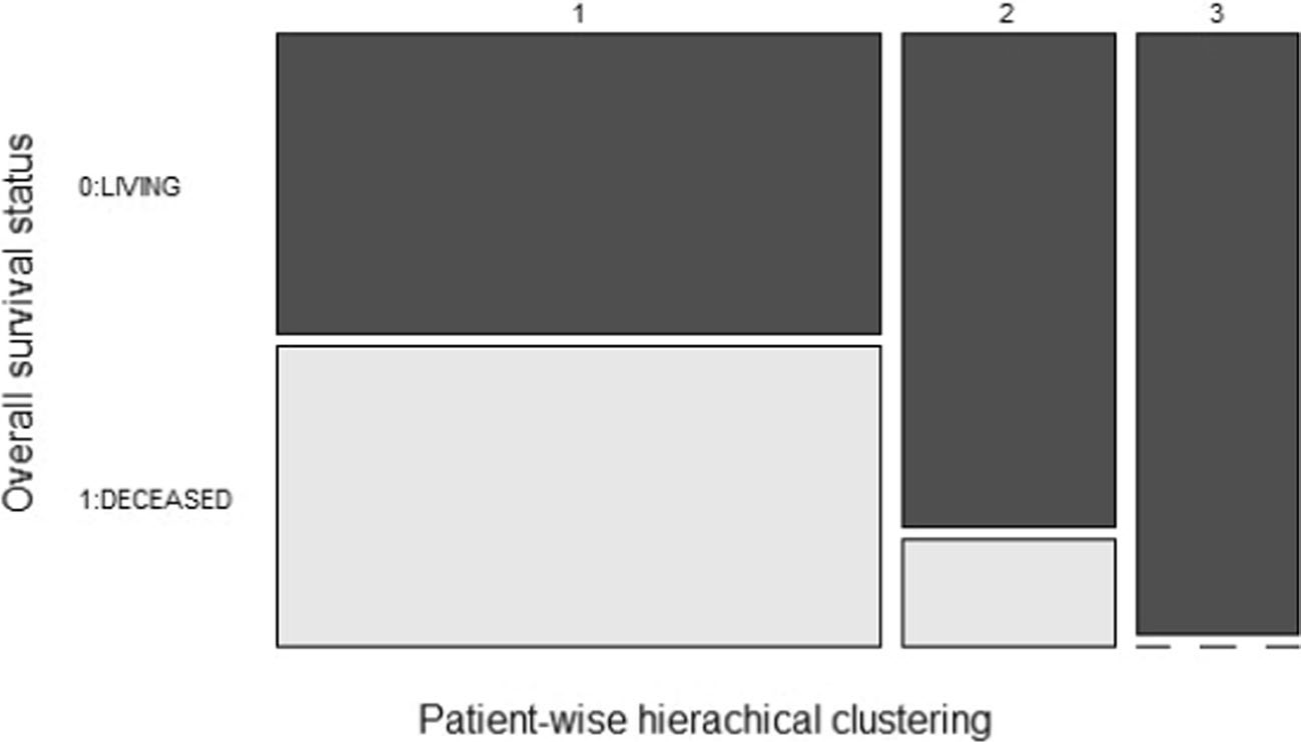
Mosaic plot of the three clusters emerged from patient-wise hierarchical clustering compared with the patient overall survival status annotations

Similarly, considering the available median follow-up of 39.3 months, it is relevant to assess prognostic insights arising from each cluster by means of the overall survival status and months annotations (Fig. 7).

From these comparisons we can notice that all patients in our third cluster have steroid phenotype low without proliferation and are living. Conversely, the first and biggest cluster has a majority of steroid phenotype high cases (82%), half with and half without proliferation; out of them, only 24 are still living, with more than 20% annotated with a recurrence event, while 24 patients are deceased, 14 within the first 2 years after diagnosis. Eventually, the second cluster, despite its heterogeneous phenotype, includes only 3 cases with increased proliferation, of which one is deceased and another one is recurred after 5 years; overall there are only 3 deceased patients, all having high steroid phenotype, while the high majority of this cluster patients is living and disease-free. Thus, the smaller second and third cluster patients, sharing wild type proliferation levels, show better expected prognoses compared with the first cluster patients.

This use case demonstrates the easiness of combining remote and local (also possibly private) data, and of using remote processing for a computationally intensive task, like a join operation on large datasets. It proves also the advantage of locally retrieving only the computed results, which are typically much smaller in size. Furthermore, it shows the usefulness of the *filter_and_extract()* function, which allows selecting from a resulting dataset and importing in the R environment only the specific data of interest for the next processing. The so-obtained GRanges format can indeed be easily manipulated and analyzed also with many R functions from different packages. The use case shows how to use several of them for an effective local exploration of data and visualization of the results from the remote processing of a large dataset, supporting also their clinical/biological assessment and interpretation.

### Use case 3: Identification of transcription factor high accumulation DNA zones

In this last use case, we report a more complex computational workflow (schematically illustrated in Additional file 5) to stress the usefulness of RGMQL and remote processing in performing complex queries on large datasets; RGMQL provided results can then be straightforwardly analyzed in the local R environment with full interoperability. Particularly, as an example we illustrate how to identify transcription factor (TF) High Occupancy Target (HOT) regions [50–52]. TFs are proteins that control the rate of transcription of genetic information from DNA to RNA, by binding to specific DNA sequences. Investigating HOT DNA regions, bound by many different transcription factors, is crucial to understand cancer genesis and develop new targeted therapies. RGMQL can automate all the steps needed to identify TF high accumulation DNA zones, interoperably cooperating with the TFHAZ Bioconductor package [53].

ChIP-seq data describes protein interactions with the DNA, including those of transcription factors. Their processing is usually specialized in identifying broad domains (covering wider DNA regions) or narrow peaks (limited to local spikes); both of them are worthy to be investigated to find HOT regions. Thus, we consider both BROAD PEAK and NARROW PEAK datasets released from the ENCODE consortium [5], available in the remote GMQL curated repository with 2,136 and 11,468 samples, respectively. Using the *filter()* function, we select ChIP-seq data of high quality (*peaks* and *optimal idr thresholded peaks*) focusing on the human embryonic stem cell line H1-hESC, and we group together the so-obtained samples in a single dataset through the *union()* function:

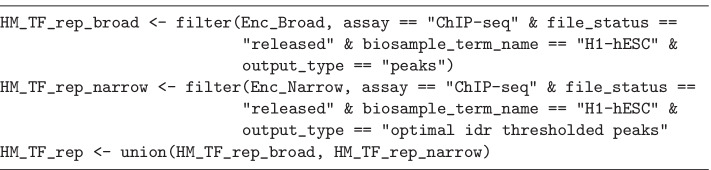



Then, we filter out all samples subjected to pharmacological treatment or annotated with quality issues, and we further use the *filter()* function to discard all samples regarding histone modifications:

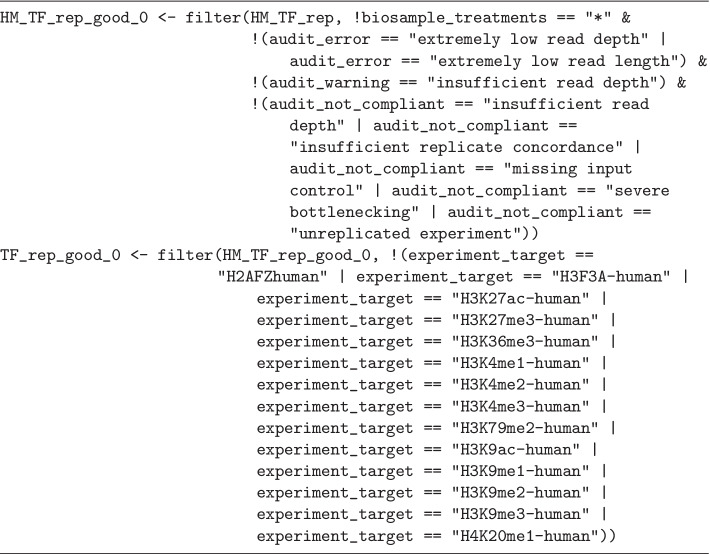



Also, we add the length of each DNA region in each sample as a new region attribute through the *regions_update* parameter of the *select()* function; then, for each sample we compute the number of regions and the sum of their lengths, and store them as sample metadata using the *extend()* function:

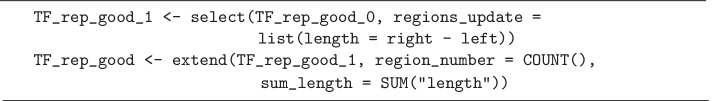



Once our main GMQLDataset *TF_rep_good* (including DNA regions that are binding sites of transcription factors) is ready, we use it in two independent but related processings. The first one is needed to extract a threshold able to identify transcription factor bound DNA regions of interest. After aggregating all samples in a single sample through the *aggregate()* function (notice that ’*biosample_term_name*’ is uniquely associated with the value ’H1-hESC’ by dataset construction), we order regions by ascending values of their length through the *arrange()* function; then, we execute the RGMQL query to materialize its result and download it in the local R environment as a GRangesList:

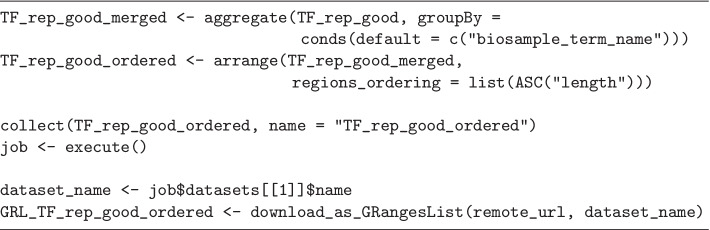



We further process such result locally to extract from the regions, ordered by their length, the index of their 95th percentile: our threshold is thus the length of the region placed at the so-obtained index. This threshold is useful to distinguish DNA regions worthy to be further examined from too wide regions, having length over the threshold. Such wide regions are indeed outliers that can affect HOT region detection, since their big length can biasedly increase the number of TFs that bind the region.

At this point, going back to the RGQML remote processing and to our main GMQLDataset *TF_rep_good*, we perform a second processing; first, it selects the DNA regions to be further examined based on the just computed threshold; then, it adds all the attributes needed for the following HOT region detection. With the *filter()* function we select as regions of interest only those with a length smaller than the just computed superior threshold, but at least greater than a given inferior threshold (e.g., 100 bases). Then, through the *extend()* function we create new metadata attributes (*region_number_filtered* and *sum_length_filtered*) by computing, for each sample, the current number of contained DNA regions and the sum of their lengths, respectively:

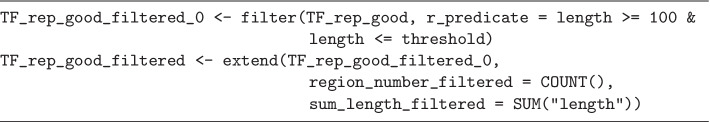



Following, we combine samples of experiments having the same target TF, using the *cover()* function with ’*experiment_target*’ as grouping parameter. Then, through the *regions_update* parameter of the *select()* function we update the values of the region attribute *length* for the obtained combined regions. Also, using the *extend()* function we create new useful metadata attributes for each of the so-obtained samples: the number of so-obtained combined regions (which have the same TF as target) and the min, max and sum of their lengths:

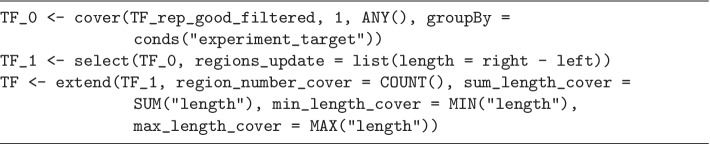



The so-obtained dataset *TF* is downloaded in the current local R environment as a GRangesList object to be straightforwardly further processed and analyzed. From the GRangesList, a GRanges object *GR_H1_hESC* is extracted, including a flatten list of all genomic regions (ranges) coming from all the *TF* samples, together with a single metadata, i.e., the annotation of the TF binding each region. *GR_H1_hESC* contains 344,556 ranges from different chromosomes, annotated with 28 different transcription factors. It is analyzed with the following functions of the TFHAZ package [53]. First, the *accumulation()* function is used to compute the accumulation vector on all chromosomes of interest, i.e., the number of transcription factors binding each base of a chromosome; this is done here below for chromosome 21 as an example. Then, the *high_accumulation_zones()* function is applied on the accumulation vector (*TF_acc_21_w_0*) to extract HOT zones, which have greater number of TF binding regions, according to the ’overlaps’ identification method. This method uses a single-base local approach and considers all and only the DNA bases of the TF accumulation vector to compute the threshold (as mean accumulation plus twice standard deviation) needed to identify the HOT zones.

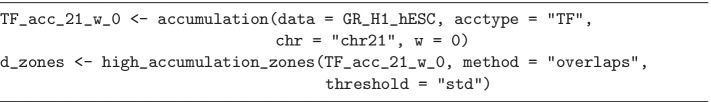



**Fig. 8:**
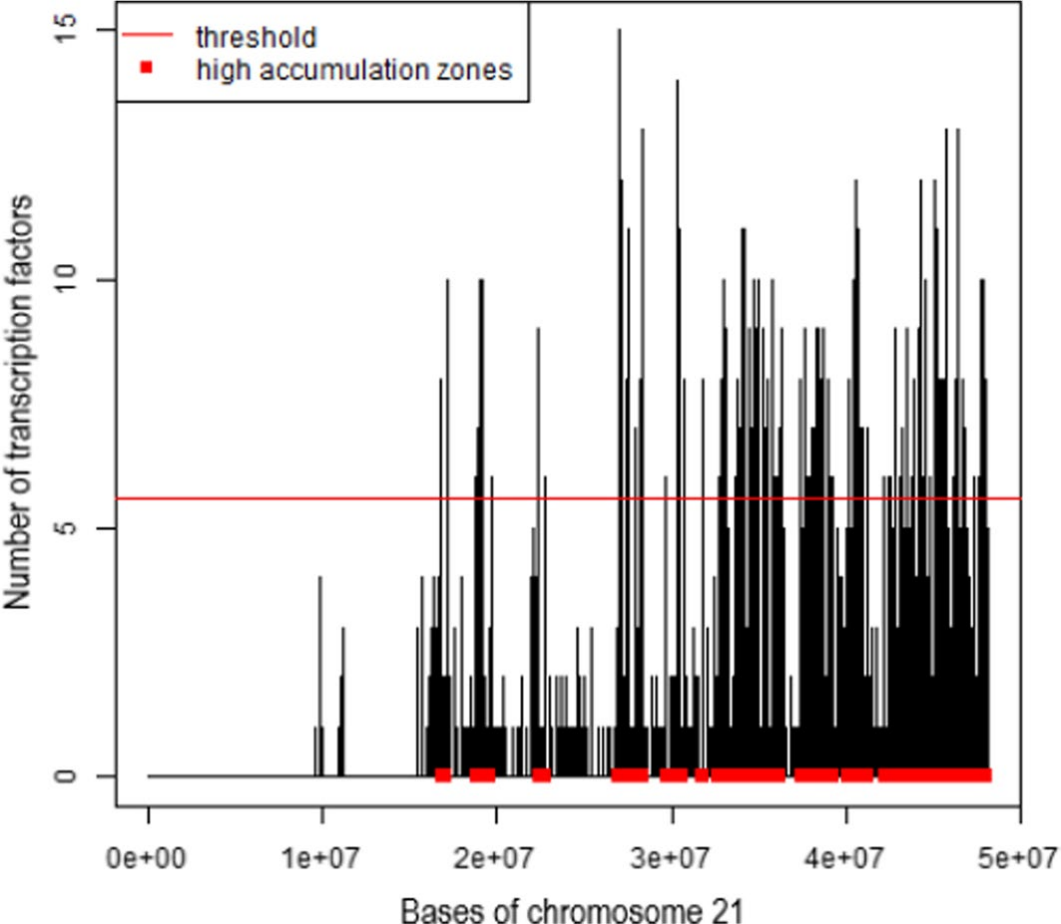
Plot of the transcription factor accumulation for chromosome 21 and of the 186 HOT zones (in red) identified according to the found accumulation threshold 5.6 (red line)

From this analysis, in chromosome 21 we find 186 HOT DNA regions, each bound by more than 5 transcription factors according to the threshold of 5.6 computed by the *high_accumulation_zones()* function. Figure 8 shows that the HOT regions obtained are mainly present in the second half of the chromosome 21 and absent from its initial portion.

The computational workflow discussed in this use case highlights the precious role of RGMQL in simplifying complex analysis such as the considered one. It allows performing the entire processing within the R environment, but without requiring consumption of local computational resources: indeed it takes advantage of the scalability and parallel computing offered by GMQL. This result can not be achieved by using only the GMQL system, or only the TFHAZ package together with other available R/Bioconductor packages. Indeed, in processing omics data, also big, RGMQL profits of both the facilities of the R environment and of the data and computational resources offered by the GMQL ecosystem. Therefore, once again, RGMQL demonstrates to play a key role in providing both a useful interactive procedural approach, typical of the bioinformatics research, and scalable performance.

## Conclusions

As R is able to interface with a variety of other languages to take advantage of well-established and state-of-the-art algorithms and protocols, similarly the R/Bioconductor RGMQL package is designed to be fully interoperable with other R packages, as well as with GMQL. Indeed, it provides the query expressiveness, computational efficiency and scalability of GMQL in the R/Bioconductor environment. Specialized RGMQL functions can extract, combine and manipulate omics big data and their metadata from different and differently localized sources. To this aim, RGMQL extends the most used genomic data structures and processing functions, and is completely integrated within the R/Bioconductor framework.

As proven in our three examples of biologically relevant use cases, RGMQL can leverage on public data hosted in the remote GMQL repository, and take advantage at runtime of the most suitable processing mode. This can be chosen according to the analysis workflow of interest and the location of the involved data, and it can be easily changed along the workflow. RGMQL further key added values are indeed the flexibility and easiness of use. Switching between local and remote processing with a single line of code, users can benefit from outsourcing the computational burden to the GMQL engine. Involved data are always moved automatically, when needed, thanks to the implemented data distribution transparency. Additionally, RGMQL allows merging not only remote and local public data, but even proprietary data: also in case of remote processing they are loaded in a private area of the remote repository, accessible through authenticated login only.

Furthermore, RGMQL is able to guarantee the FAIR principles of findability, accessibility, interoperability and reusabilty, both at the data and at the implementation level. Hence, RGMQL is definetely a versatile and valuable ally in the R/Bioconductor-based genomic research, in particular for scalable omics data tertiary investigations.

## Availability and requirements

- Project name: RGMQL
- Project home page: on GitHub: https://github.com/DEIB-GECO/RGMQL on Bioconductor: https://www.bioconductor.org/packages/release/bioc/html/RGMQL.html
- Operating system(s): Platform independent
- Programming languages: R and Scala/Java
- Other requirements: R ($\ge$ 3.4.2), Java ($\ge$ 1.8)
- License: Artistic-2.0
- Any restrictions to use by non-academics: None

## Supplementary information

**Additional file 1**. Supplementary figure of the use case 1, showing the counts of mutated genes for each KIRC patient younger than 65 years

**Additional file 2**. Supplementary figure of the use case 1, showing the top 20 genes by number of mutations across the 217 patients under analysis, orderly and proportionally plotted horizontally by their gene length, from left (VHL - 12,036 bp) to right (PCDHA@ - 226,209 bp)

**Additional file 3**. Flowchart of the main steps of use case 2. As illustrated, starting from both local and remote gene expression datasets, a RGMQL mixed processing first joins the two datasets remotely, then downloads and processes the result locally. After the generation of a samples-per-genes dataset, also the main phases of local post-processing with clustering analysis are depicted

**Additional file 4**. Supplementary figure of the use case 2, showing the optimal number of clusters based on the average silhouette width

**Additional file 5**. Flowchart of the main steps of use case 3. As illustrated, after RGMQL remote pre-processing of the ENCODE ChIP-seq datasets of interest, two independent but related RGMQL processing are performed. The first one computes the threshold needed to select the DNA regions on which the transcription factor (TF) accumulation must be assessed. The second one uses the threshold to select such regions, and process them up to find HOT DNA zones by cooperating with the TFHAZ Bioconductor package

## Data Availability

RGMQL package, documentation and code are freely available at https://github.com/DEIB-GECO/RGMQL and https://www.bioconductor.org/packages/release/bioc/html/RGMQL.html The datasets analysed during the current study are publicly available in the GMQL repository at http://www.gmql.eu/.

## Abbreviations

1KGP: 1000 Genomes Project
ACC: Adrenocortical carcinoma
API: Application Programming Interface
BED: Browser Extensible Data
CRAN: Comprehensive R Archive Network
DAG: directed acyclic graph
ENCODE: Encyclopedia of DNA Elements
GDM: Genomic Data Model
GMQL: GenoMetric Query Language
HDFS: Hadoop File System
HOT: High Occupancy Target
KIRC: Kidney Renal Clear Cell Carcinoma
NGS: Next Generation Sequencing
Pcdhs: protocadherins
RefSeq: Reference Sequence
TCGA: The Cancer Genome Atlas
TF: Transcription factor

## Footnotes

1 https://www.bioconductor.org/news/bioc_3_14_release/.
