# Supplementary figures and images for: RGMQL: scalable and interoperable computing of heterogeneous omics big data and metadata in R/Bioconductor
Source: BMC Bioinformatics. 2022 Apr 7;23:123. doi: 10.1186/s12859-022-04648-4 (PMC8991469; doi:10.1186/s12859-022-04648-4)

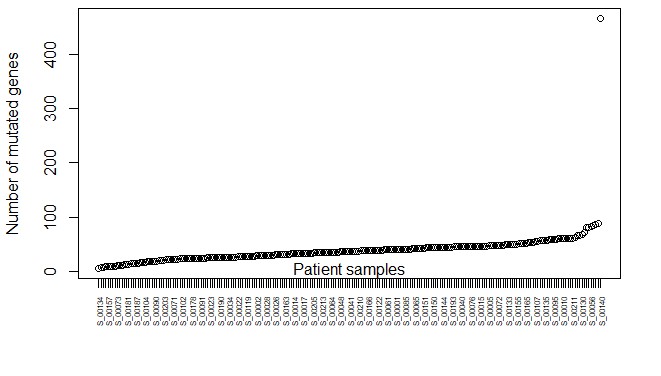

Supplement: Supplementary file 1 — Additional file 1. Supplementary figure of the use case 1, showing the counts of mutated genes for each KIRC patient younger than 65 years [file 12859_2022_4648_MOESM1_ESM.jpg]

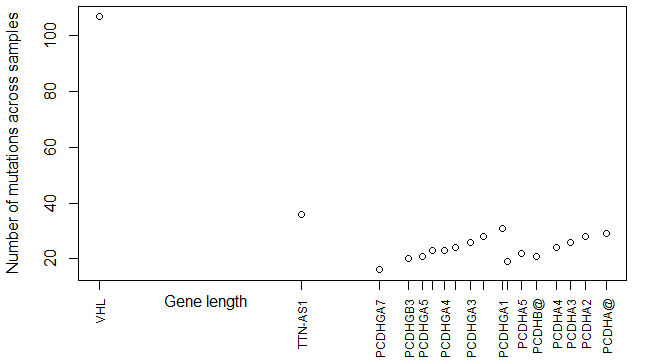

Supplement: Supplementary file 2 — Additional file 2. Supplementary figure of the use case 1, showing the top 20 genes by number of mutations across the 217 patients under analysis, orderly and proportionally plotted horizontally by their gene length, from left (VHL - 12,036 bp) to right (PCDHA@ - 226,209 bp) [file 12859_2022_4648_MOESM2_ESM.png]

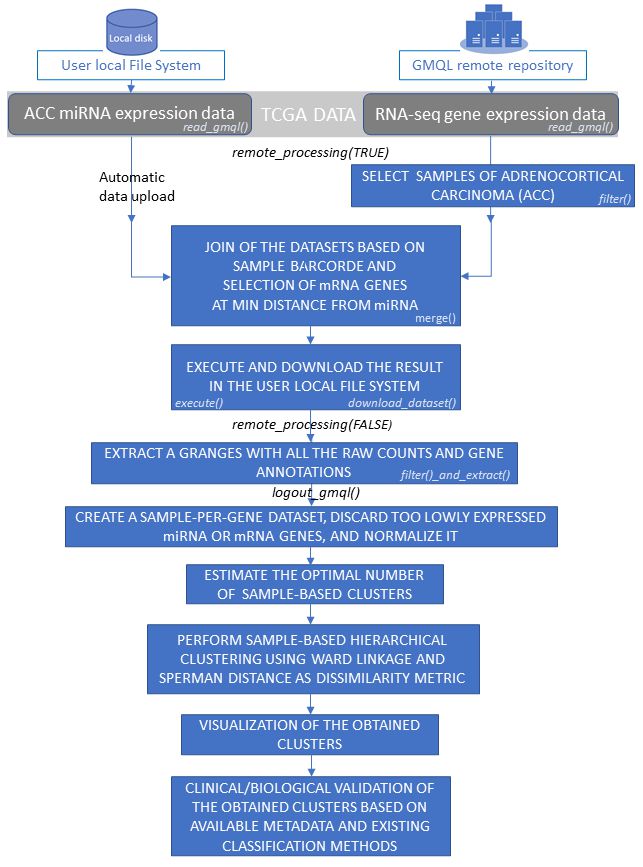

Supplement: Supplementary file 3 — Additional file 3. Flowchart of the main steps of use case 2. As illustrated, starting from both local and remote gene expression datasets, a RGMQL mixed processing first joins the two datasets remotely, then downloads and processes the result locally. After the generation of a samples-per-genes dataset, also the main phases of local post-processing with clustering analysis are depicted [file 12859_2022_4648_MOESM3_ESM.jpg]

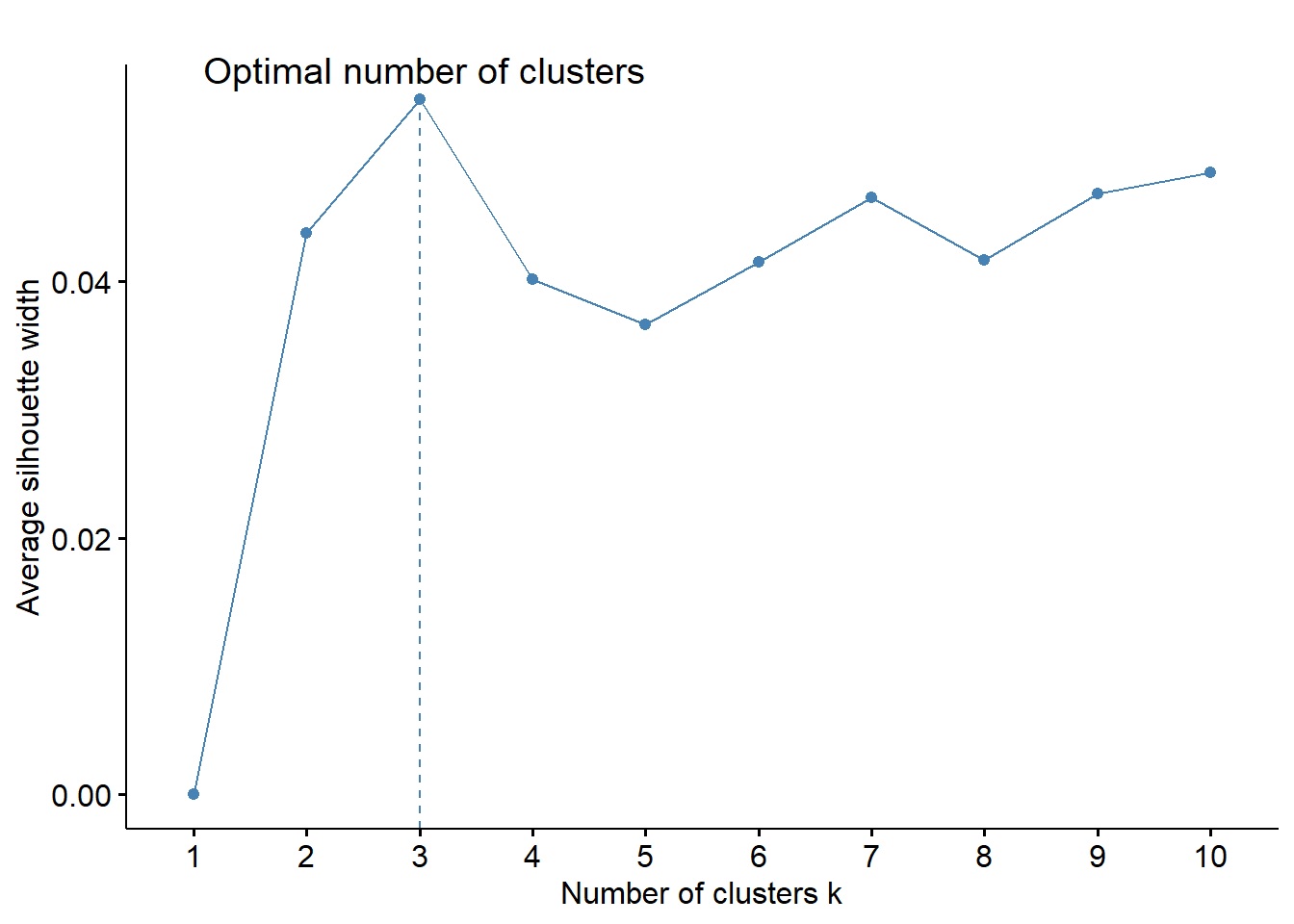

Supplement: Supplementary file 4 — Additional file 4. Supplementary figure of the use case 2, showing the optimal number of clusters based on the average silhouette width [file 12859_2022_4648_MOESM4_ESM.jpg]

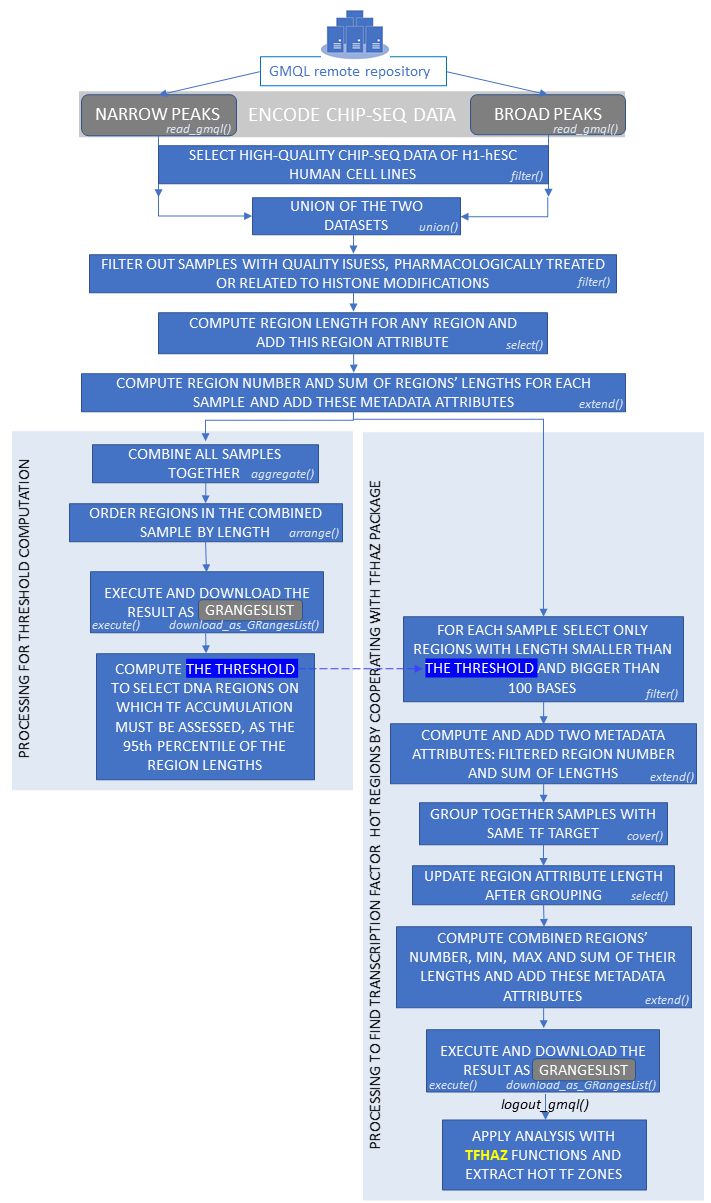

Supplement: Supplementary file 5 — Additional file 5. Flowchart of the main steps of use case 3. As illustrated, after RGMQL remote pre-processing of the ENCODE ChIP-seq datasets of interest, two independent but related RGMQL processing are performed. The first one computes the threshold needed to select the DNA regions on which the transcription factor (TF) accumulation must be assessed. The second one uses the threshold to select such regions, and process them up to find HOT DNA zones by cooperating with the TFHAZ Bioconductor package [file 12859_2022_4648_MOESM5_ESM.jpg]
